# Drug-Drug Interactions in the Management of Patients With Pulmonary Arterial Hypertension

**DOI:** 10.1016/j.chest.2022.06.042

**Published:** 2022-07-14

**Authors:** Sheryl Wu, Heather B. Hoang, Jenny Z. Yang, Demosthenes G. Papamatheakis, David S. Poch, Mona Alotaibi, Sandra Lombardi, Cynthia Rodriguez, Nick H. Kim, Timothy M. Fernandes

**Affiliations:** University of California, San Diego, La Jolla, CA

**Keywords:** cytochrome P450, drug interactions, PAH, pulmonary arterial hypertension, CCB, calcium channel blocker, cGMP, cyclic guanosine monophosphate, CYP450, cytochrome P450, ERA, endothelin receptor antagonist, ET_A_, endothelin A, ET_B_, endothelin B, NO, nitric oxide, OATP, organic anion-transporting polypeptide, PAH, pulmonary arterial hypertension, PDE5, phosphodiesterase 5, sGC, soluble guanylate cyclase

## Abstract

The management of pulmonary arterial hypertension (PAH) has become more complex in recent years because of increased pharmacotherapy options and longer patient survival with increasing numbers of comorbidities. As such, more opportunities exist for drug-drug interactions between PAH-targeted medications and medications potentially used to treat comorbid conditions. In this review, we provide an overview of pharmaceutical metabolism by cytochrome P450 and discuss important drug-drug interactions for the 14 Food and Drug Administration-approved medications for PAH in the nitric oxide (NO), endothelin, and prostacyclin pathways. Among the targets in the NO pathway (sildenafil, tadalafil, and riociguat), important interactions with nitrates, protease inhibitors, and other phosphodiesterase inhibitors can cause profound hypotension. In the endothelin pathway, bosentan is associated with more drug interactions via CYP3A4 inhibition; macitentan and ambrisentan have fewer interactions of note. Although the parenteral therapies in the prostacyclin pathway bypass significant liver metabolism and avoid drug interactions, selexipag and oral treprostinil may exhibit interactions with CYP2C8 inhibitors such as gemfibrozil and clopidogrel, which can raise drug levels. Finally, we provide a framework for identifying potential drug-drug interactions and avoiding errors.

In the 1980s, before the advent of pulmonary arterial hypertension (PAH)-targeted medical therapies, PAH (then referred to as *primary pulmonary hypertension*) carried a terrible long-term prognosis, with only a 68% 1-year patient survival rate and a 34% 5-year patient survival rate.^1^ At that time, PAH was a disease of the young (mean age, 36 years) and women (1.7:1 female to male ratio) with few comorbidities. Over the past 25 years, PAH has evolved from a disease with no specific treatments and a dismal prognosis to a disease with three main treatment pathways and significantly improved life expectancy. In the more contemporaneous Registry to Evaluate Early And Long-term PAH Disease Management (REVEAL), the mean age at PAH diagnosis increased to 50.1 years and more comorbidities were seen among patients living with PAH.^2^ Today, more than two-thirds of all patients with idiopathic PAH have significant comorbidities (with one in seven having four or more comorbid conditions), making this patient population more complex to treat than in the past.^3^

Not only have patients become more complicated, but the options for medical management of PAH also have increased. Fourteen medications are now approved by the Food and Drug Administration for PAH used in multiple combinations.^4^ The currently approved PAH-targeted therapies act on three main pathways: the nitric oxide (NO) pathway (including the phosphodiesterase 5 [PDE5] inhibitors tadalafil and sildenafil and the soluble guanylate cyclase [sGC] stimulator riociguat), the endothelin pathway (bosentan, ambrisentan, and macitentan), and the prostacyclin pathway (including the various formulations of the prostacyclin analogs epoprostenol, iloprost, and treprostinil and a prostacylin-receptor agonist, selexipag). Given the increasing complexity of patients with PAH in terms of comorbidities and disease-specific management, numerous opportunities exist for drug-drug interactions between PAH medications and other drugs or supplements for comorbid conditions.

Cytochrome P450 (CYP450) is an enzyme that plays a fundamental role in the metabolism of medications.^5^ Drugs with CYP450 activity may be inhibitors, inducers, substrates, or a combination thereof for a specific CYP450 enzymatic pathway that can change the metabolism of concurrently administered medications. Inhibitors are substances that reduce an enzymatic pathway of CYP450 and may cause increased concentrations of other drugs metabolized by the same pathway, resulting in drug toxicity. Inducers are substances that induce an enzymatic pathway of CYP450, which may increase metabolism of other drugs by the same pathway, leading to subtherapeutic drug levels and treatment failure.^6^ Although more than 50 isoforms of CYP450 have been discovered, six of them (CYP3A4, CYP2D6, CYP1A2, CYP2C9, and CYP2C19) metabolize 90% of drugs, with the two most significant enzymes being CYP3A4 and CYP2D6.^7^

As our understanding of CYP450 metabolism continues to grow, new agents undergo extensive drug interaction studies performed before becoming available. However, not all agents have been tested in combination, and at times drug interactions are hypothesized based on known metabolic pathways. As a result, drug-drug interactions may include the magnification of known potential adverse effects. Not all cytochrome P450-mediated drug interactions are clinically significant, and thus may or may not require dosage adjustments. Active drug transporters, P-glycoprotein, and human organic anion-transporting polypeptides (OATPs), also play an important role in drug elimination and affect the bioavailability of a number of drugs by controlling their movement into and out of cells.^8^ Refer to Table 1 for commonly used medications with known CYP450, P-glycoprotein, and OATP activity. Knowledge of the drugs metabolized by CYP450 enzymes, active drug transporters, and the most potent inhibiting and inducing drugs can help to minimize the possibility of adverse drug reactions and interactions or therapeutic failures.^9^

**Table 1:** Common Sites of Drug Metabolism and Interactions

CYP3A4	
Inhibitors	Azole antifungals, cobicistat, ritonavir, amiodarone, macrolide antibiotics (erythromycin), cyclosporine, diltiazem, verapamil
Inducers	Carbamazepine, phenytoin, rifampin, St. John’s wort, smoking
Substrates^a^	Hormonal contraceptives, HmG-CoA reductase inhibitors (primarily simvastatin), colchicine
CYP2C8	
Inhibitors	Strong: gemfibrozil; moderate: clopidogrel, deferasirox, leflunomide, teriflunomide; weak: abiraterone, montelukast, trimethoprim
Inducer	Rifampin
Substrates	Chloroquine, paclitaxel, repaglinide, rosiglitazone
CYP2C9	
Inhibitors	Ritonavir, amiodarone, fluconazole, sulfamethoxazole-trimethoprim
Inducer	Rifampin
Substrate	Warfarin, bosentan, losartan, naproxen
CYP2C19	
Inhibitors	Omeprazole, fluconazole, ketoconazole, isoniazid
Inducers	Carbamazepine, phenytoin, rifampin
Substrates	Clopidogrel, omeprazole, citalopram
CYP1A1	
Inducer	Smoking
P-glycoprotein efflux pump	
Inhibitors	Cyclosporine, ketoconazole, ritonavir, amiodarone, clarithromycin, propafenone, quinidine, ranolazine, verapamil
Inducer	St. John’s wort
Substrate	Digoxin
OATP hepatic transporter	
Inhibitors	Gemfibrozil, cyclosporine

Inhibitors: Expect increase in substrate plasma concentration; Inducers: Expect decrease in substrate plasma concentration; Substrates: Metabolism will be affected by inhibitors or inducers, resulting in increase or decrease in plasma concentrations, respectively. OATP = organic anion-transporting polypeptide

a More than 1,000 medications are metabolized by CYP3A4. This list identifies drugs commonly affected by PAH medications.

**Table 2:** Clinically Established and Other Potentially Significant Drug Interactions: Endothelin Receptor Antagonists

PAH Drug	Interacting Drug	Mechanism	Effect	Recommendation
Bosentan	Glyburide	Additive hepatotoxicity	Increased incidence of elevated aminotransferases	Contraindicated
	Hormonal contraceptives	CYP3A4 induction by bosentan, reducing plasma concentration of hormonal contraceptives	Unreliable contraception	Counsel patients to use additional method of contraception
	HMG CoA reductase inhibitors	CYP2C9 induction by bosentan, decreasing HMG CoA reductase inhibitor levels	Simvastatin levels reduce 50%	Monitor cholesterol levels
	Cyclosporine	CYP3A4 and OATP inhibition by cyclosporine, increasing bosentan concentrations CYP3A4 induction by bosentan, decreasing cyclosporine concentration	Bosentan levels increased fourfold	Contraindicated
	Amiodarone, fluconazole	CYP3A4 and CYP2C9 inhibition, increasing bosentan concentrations	Likely effect, not quantified in the literature	Not recommended
	Ketoconazole	CYP3A4 inhibition, increasing bosentan concentrations	Bosentan 125 mg twice daily administered with ketoconazole increased bosentan plasma concentration by 100%	No dose adjustment necessary; monitor for increased effects
	Ritonavir (including nirmatrelvir/ritonavir for COVID-19)	CYP3A4 inhibition, increasing bosentan concentrations	Fivefold increase in bosentan exposure	Discontinue bosentan at least 36 h before initiation of ritonavir; may resume bosentan at recommended initial dose once daily after at least 10 d after the initiation of ritonavir
	Phenytoin, rifampin	CYP3A4 and CYP2C9 induction, decreasing bosentan concentration	Rifampin decreased bosentan concentrations by more than 50%	Recommend that liver function tests be measured weekly for the first 4 w; normal liver function monitoring may be conducted subsequently
Ambrisentan	Cyclosporine	CYP3A4 and P-glycoprotein inhibition, increasing ambrisentan concentration	Twofold increase in ambrisentan exposure	Dose reduce ambrisentan to 5 mg once daily
Macitentan	Amiodarone, fluconazole	CYP3A4 and CYP2C9 inhibition, increasing macitentan concentration	Predicted to increase macitentan exposure fourfold	Macitentan increase not clinically relevant; use with caution and monitor for increased side effects
	Ketoconazole	CYP3A4 inhibition, increasing macitentan concentration	Macitentan exposure doubled	Not clinically relevant; dose adjustments not necessary
	Rifampin, carbamazepine	CYP3A4 induction, decreasing macitentan concentration	Macitentan exposure decreased by 80% when given with rifampin 600 mg daily	Avoid because of reduced efficacy; dosing recommendations not established

PAH = pulmonary arterial hypertension.

**Table 3:** Clinically Established and Other Potentially Significant Drug Interactions: NO Pathway

PAH Drug	Interacting Drug	Mechanism	Effect	Recommendation
Sildenafil	Nitrates	Additive potent vasodilation	Profound systemic hypotension	Contraindicated; if necessary, at least 24 h of separation between the last dose of sildenafil and nitrate administration is recommended
	Bosentan	CYP3A4 induction, decreases sildenafil levels; sildenafil is a substrate of CYP3A4, competing with metabolism of bosentan (also CYP3A4 substrate), resulting in increased bosentan levels	50% reduction in the serum concentration of sildenafil and 50% increase in bosentan concentration	No dose adjustments necessary; however, no benefit on exercise capacity demonstrated when used concomitantly
	Ritonavir (including Paxlovid for COVID-19)	CYP3A4 inhibition, increases sildenafil levels	Sildenafil exposure increased up to 1,000%	Contraindicated
	Cobicistat, ketoconazole	Potent CYP3A4 inhibition, increases sildenafil levels	Expect similar effects to ritonavir	Not recommended
	St. John’s wort	CYP3A4 induction, reduces sildenafil levels	Threefold increase in sildenafil clearance	Efficacy may be reduced; may consider dose increase of sildenafil under close monitoring
	Phenytoin, rifampin,	CYP3A4 induction, reduces sildenafil levels	Expect significant decreases in sildenafil levels	Not recommended; may result in near-complete clearance of sildenafil
Tadalafil	Nitrates	Additive potent vasodilation	Profound systemic hypotension	Contraindicated; if necessary, at least 48 h of separation between the last dose of sildenafil and nitrate administration is recommended
	Ketoconazole	CYP3A4 inhibition, increases tadalafil levels	Ketoconazole 400 mg daily with a single 20-mg tadalafil dose increased the tadalafil exposure by 312%; ketoconazole 200 mg daily increased tadalafil exposure by 107%	Avoid use
	Ritonavir (including Paxlovid for COVID-19)	CYP3A4 inhibition, increases tadalafil levels	Inhibits tadalafil in a time dependent manner	Avoid use of tadalafil during initiation of ritonavir; consider stopping at least 24 h before ritonavir initiation; resume tadalafil at 20 mg once daily after 1 w of ritonavir initiation
	Rifampin	CYP3A4 induction, reduces tadalafil	Tadalafil exposure reduced by 88%	Not recommended in patients taking long-term rifampin
Riociguat	Nitrates	Additive potent vasodilation	Hypotension leading to syncope	Contraindicated; data not available to decide dosing
	Antacids	Increases pH of stomach contents	Reduces solubility of riociguat up to 34%	Do not use antacids within 1 h of riociguat
	Ketoconazole	CYP3A4 and P-glycoprotein inhibition, increases riociguat levels	Riociguat exposure increased by 150%	Consider riociguat initiation dose of 0.5 mg 3 times daily
	Ritonavir (including Paxlovid for COVID-19)	CYP3A4 inhibition, increases riociguat levels	Expect similar effect to ketoconazole	Avoid interaction. Select alternative COVID-19 anti-viral.
	Tobacco smoke	CYP1A1 inducer, reduces riociguat levels	Plasma concentrations of riociguat in tobacco users are reduced by 50%-60% compared with non-tobacco users	Doses higher than 2.5 mg three times daily may be considered

NO = nitric oxide; PAH = pulmonary arterial hypertension.

**Table 4:** Clinically Established and Other Potentially Significant Drug Interactions: Prostacyclin Pathway Drugs

PAH Drug	Interacting Drug	Mechanism	Effect	Recommendation
Treprostinil diethanolamine (oral formulation)	Gemfibrozil	CYP2C8 inhibition, increases treprostinil diethanolamine levels	Twofold increase in treprostinil diethanolamine concentrations	Reduce the starting dose of treprostinil diethanolamine to 0.125 mg twice daily and increase by 0.125-mg twice daily increments not more frequently than every 3-4 d
Selexipag	Clopidogrel	Moderate CYP2C8 inhibition, increases selexipag levels	Approximately threefold increase in selexipag concentrations	Reduce dose of selexipag to once daily
	Leflunomide	Moderate CYP2C8 inhibition, active metabolite teriflunomide increases selexipag levels	Expect similar effect to clopidogrel	Dose reduce selexipag to once daily
	Deferasirox	Moderate CYP2C8 inhibition, increases selexipag levels	Expect similar effect to clopidogrel	Dose reduce selexipag to once daily
	Gemfibrozil	Strong CYP2C8 inhibition, increases selexipag levels	11-fold increase in selexipag concentrations	Strong inhibitors are contraindicated
	Rifampin	CYP3A4 induction, decreases selexipag concentration	Decrease in active metabolite of selexipag by 50%	Dose of selexipag should be doubled when starting rifampin and then reduced when rifampin is stopped

PAH = pulmonary arterial hypertension.

Multiple drugs may target each of the three pathways for PAH treatment, and not all the medications in a class share the same drug-drug interactions. Providers should be aware of the potential drug-drug interactions that may affect patient care detrimentally. Herein, we review each of the three PAH treatment pathways and summarize the related drug-drug interactions commonly encountered.

### The Endothelin Pathway: Bosentan, Ambrisentan, and Macitentan

Endothelin receptor antagonists (ERAs) competitively inhibit endothelin 1 to decrease pulmonary vascular resistance. Endothelin 1 is a potent vasoconstrictor that also mediates cell proliferation, fibrosis, and inflammation. Endothelin 1 binds to endothelin receptor A (ET_A_), which leads to pulmonary vasoconstriction and smooth muscle cell proliferation, and endothelin receptor B (ET_B_), which reduces endothelin 1 and induces endothelial cell vasodilation via NO and prostacyclin release.^10^ ERAs differ in selectivity between ET_A_ and ET_B_ receptors. Ambrisentan shows 100 times greater selectivity for ET_A_ than ET_B._ Bosentan and macitentan are considered nonselective for the ET_A_ and ET_B_ receptors, with bosentan being 20 times more selective for ET_A_ than ET_B_ and macitentan being 50 times more selective for ET_A_ than ET_B_. ERAs block the activation of ET_A_ and ET_B_ receptors on endothelial or smooth muscle cells and inhibit the vasoconstriction and cellular proliferation mediated by endothelin 1.^11^

Of the three available ERAs, bosentan has the most potential for adverse events resulting from drug-drug interactions because of its side effect profile and multiple metabolism mechanisms via CYP450 isoenzymes (Table 2). Elevations in liver aminotransferase levels more than three times the upper limit of normal have been observed with bosentan use because of active transport into the liver via OATP transporters. Other drugs with known hepatotoxicity should be used with caution, especially glyburide, which is contraindicated with bosentan because of an increased risk of liver enzyme elevations.^12^ Bosentan is metabolized by CYP3A4 and CYP2C9; therefore, other medications that affect these enzymes through inhibition or induction will affect bosentan levels. Cyclosporine, a CYP3A4, and OATP inhibitor, substantially increases bosentan plasma concentrations, and concomitant use is contraindicated. Combinations of a potent CYP3A4 inhibitor, a CYP2C9 inhibitor, or both with bosentan likely will cause a significant increase in plasma bosentan levels and are not recommended. Notably, bosentan also is an inducer of CYP3A4 and CYP2C9 and can reduce plasma concentrations of coadministered drugs metabolized by these isoenzymes. Patients taking oral hormonal contraceptives, which commonly are CYP3A4 substrates, should be advised of possible decreased contraceptive efficacy, especially considering the teratogenic properties of bosentan.^13^ Warfarin is a CYP2C9 substrate; however, therapeutic doses of bosentan did not cause clinically relevant changes in international normalized ratio when used together.^14^

Ambrisentan shows the least risk for drug-drug interactions among the ERAs because metabolism is primarily through the less common pathway of hepatic glucuronidation.^13^ Ambrisentan also is a minor substrate of CYP3A4, CYP2C9, OATP, and P-glycoprotein efflux pump. However, a significant clinically relevant drug-drug interaction exists with cyclosporine.^15^ Cyclosporine increases ambrisentan exposure by twofold, which warrants a dose reduction of ambrisentan to 5 mg once daily when administered together. A prior report showed that the overall safety profile of ambrisentan was similar in the presence and absence of rifampin, and no dose adjustment of ambrisentan was required.^16^ The potential for interactions with other drugs that have activity on CYP3A4, CYP2C9, OATP, and P-glycoprotein still exists and should be considered in patients with polypharmacy and concomitant use of drugs with narrow therapeutic windows.^17^ Nasal congestion is a common adverse effect caused by ERAs, most commonly ambrisentan.^18^ Although pseudoephedrine decongestants may treat nasal congestion, providers should instruct patients to avoid such stimulants that will worsen PAH acutely via vasoconstriction.^19^

Macitentan is metabolized primarily by CYP3A4 with minor metabolism by CYP2C8, CYP2C9, and CYP2C19. Per the prescribing information, coadministration with strong CYP3A4 inhibitors should be avoided with macitentan. Prescribing information also recommends avoiding concomitant use of macitentan with moderate dual CYP3A4 and CYP2C9 inhibitors because of an approximately fourfold increase in macitentan exposure.^20^ In the presence of ketoconazole, a potent CYP3A4 inhibitor, exposure to macitentan doubles. However, the increase in macitentan levels was determined to be clinically insignificant because the levels were within the ranges observed in the single- and multiple-ascending dose studies and were well tolerated.^21^ This suggests that dose adjustments of macitentan may not be necessary when given with strong CYP3A4 inhibitors similar to ketoconazole, such as ritonavir. Data on file with Janssen Research and Development show no clinically relevant change in exposure to the active metabolite of macitentan, and caution should be used when it is coadministered with a moderate dual inhibitor of CYP3A4 and CYP2C9 (fluconazole and amiodarone).^22^ Unlike bosentan and ambrisentan, macitentan is not involved with OATP for hepatic uptake or P-glycoprotein efflux pumps, which eliminates the possibility of drug interactions at those sites.^23^ As a result, no clinically relevant differences are observed when macitentan is used concurrently with cyclosporine. The lack of involvement with OATP and P-glycoprotein also allows macitentan to have fewer hepatoxicity considerations than the other ERAs. In contrast, strong CYP3A4 inducers such as rifampin or carbamazepine have clinically significant decreases in macitentan levels; if these medications must be used, switching to ambrisentan, which does not interact significantly with rifampin or carbamazepine, could be an option. Macitentan exposure is decreased by 80% when given with rifampin. Overall, macitentan has a low-risk profile for drug-drug interactions.^24^

## The NO Pathway: PDE5 Inhibitors and Riociguat

Endothelial NO induces vasodilation in vascular smooth muscle cells and inhibits platelet aggregation through the activation of sGC and the subsequent increase in production of cyclic guanosine monophosphate (cGMP).^25^ The production of endothelial NO is reduced chronically in patients with PAH.^21^ Therapeutic agents that act on the NO pathway increase intracellular cGMP concentrations ultimately targeting vasodilation of the pulmonary vasculature and PAH symptoms. PDE5 is responsible for degradation of cGMP in pulmonary smooth muscle and also is involved in platelet aggregation.^26^ PDE5 inhibitors, such as sildenafil and tadalafil, prevent the breakdown of cGMP and demonstrate antiplatelet activity. Riociguat, a guanylate cyclase stimulator, sensitizes sGC to endogenous NO and directly stimulates sGC receptors, leading to an increase in cGMP.

Considering these mechanisms of action, concomitant use of NO agents with medications that have hypotensive or antiplatelet effects may have compounding additive adverse effects (Table 3). Studies have shown that PDE5 inhibitors generally are safe with most antihypertensives and vasodilating medications, apart from nitrates and some α-adrenergic blockers.^27^, ^28^, ^29^ Organic nitrates such as nitroglycerin, amyl nitrate, and isosorbide mononitrate are associated with significant risk of life-threatening hypotension when combined with a PDE5 inhibitor or guanylate cyclase stimulator, and therefore are contraindicated. If use of an organic nitrate is mandatory, at least 24 h of separation must elapse between the last dose of sildenafil, and at least 48 h must elapse after the last dose of tadalafil. Given the ubiquitous use of nitrates and the potential for patients with PAH to demonstrate chest pain, it is important to counsel all patients taking sildenafil, tadalafil, or riociguat to warn providers in emergency medical services to avoid nitrates. Nonuroselective α-adrenergic blockers such as doxazosin have demonstrated significant hypotension when used with sildenafil, with decreases in BP of up to 15 mm Hg systolic and 22 mm Hg diastolic.^26^ Close monitoring of patients receiving α-adrenergic blockers with NO agents is recommended.

Although the PDE5 inhibitors sildenafil and tadalafil have similar efficacy and safety profiles, they differ in drug-drug interactions because of pharmacokinetic attributes. Sildenafil is highly metabolized by the cytochrome P450 isoenzyme CYP3A4, with metabolism by CYP2C9 to a lesser extent.^30^ Clearance of sildenafil is reduced when used concurrently with other CYP3A4 inhibitors, leading to higher plasma concentrations and increased severity of adverse effects such as pronounced hypotension, syncope, hearing and vision loss.^29^ Coadministration of sildenafil for PAH therapy with potent CYP inhibitors such as ritonavir and cobicistat is contraindicated. Ritonavir inhibits both sites of sildenafil metabolism at CYP3A4 and CYP2C9, resulting in a substantial increase of sildenafil levels.^31^ A single dose of sildenafil 100 mg with ritonavir 500 mg increased sildenafil exposure by 1000%. Cobicistat and ketoconazole are equally potent CYP3A4 inhibitors to ritonavir with no inhibition activity at CYP2C9, yet theoretically carry similar concerns for increase in sildenafil concentrations.^32^^,^^33^ It is important to recognize these interactions because ritonavir and cobicistat are used as inhibitors in combination medications for COVID-19 and HIV: nirmatrelvir/ritonavir, atazanavir/cobicistat, cobicistat, darunavir/cobicistat, darunavir/cobicistat/tenofovir alafenamide/emtricitabine, elvitegravir/cobicistat/emtricitabine/tenofovir alafenamide, and elvitegravir/cobicistat/emtricitabine/tenofovir. Other CYP2C9 inhibitors do not have clinically established effects on sildenafil levels.^30^ Sildenafil also is substantially sensitive to CYP3A4 inducers, which decrease efficacy of sildenafil when coadministered. An approximately threefold increase in clearance of sildenafil was observed when given with St. John’s wort, a moderate CYP3A4 inducer. Therefore, rifampin and phenytoin, potent CYP3A4 inducers, are expected to cause extensive decreases in sildenafil concentrations that necessitate a change in therapy.

One combination in PAH treatment is the use of bosentan (a CYP3A4 inducer and substrate) with sildenafil, which together demonstrated a 50% reduction in the serum concentration of sildenafil and a 50% increase in bosentan concentration via competitive inhibition. Although this reduction in serum concentration of sildenafil is not considered clinically significant, the sildenafil plus bosentan combination failed to meet the primary endpoint in the Effects of Combination of Bosentan and Sildenafil Versus Sildenafil Monotherapy on Morbidity and Mortality in Symptomatic Patients With Pulmonary Arterial Hypertension - A Multicenter, Double-blind, Randomized, Placebo-controlled, Parallel Group, Prospective, Event Driven Phase IV Study (COMPASS-2) trial, and this interaction may have contributed to this outcome.^34^ If bosentan and sildenafil are used together, patients may need to be monitored for increased bosentan adverse effects such as headache, hypotension, postural hypotension, and nasal congestion.

Compared with sildenafil, tadalafil has a longer half-life and is a less sensitive CYP3A4 substrate.^35^ If ritonavir must be used, it is advised to stop tadalafil at least 24 h before starting ritonavir because of the risk of hypotension at higher concentrations of tadalafil. As soon as ritonavir is at a steady state, approximately 1 week after initiation, tadalafil may be resumed at a lower dose of 20 mg daily to assess for tolerance before increasing to 40 mg once daily. Of note, long-term use of tadalafil with potent CYP3A4 inducers such as rifampin is not recommended. Tadalafil exposure when given with rifampin was reduced by 88%.^36^ Tadalafil also is not metabolized by CYP2C9. In contrast to sildenafil, when used in combination with bosentan, tadalafil has no clinical effect on plasma concentrations of bosentan. Therefore, tadalafil is less likely to be subjected to drug-drug interactions than sildenafil.

Riociguat use with other NO agents, including PDE inhibitors (both PDE5 inhibitors and other selective phosphodiesterase inhibitors such as theophylline or dipyridamole), soluble guanylate cyclase stimulators, and nitrates, is contraindicated because of significant hypotensive effects. When switching between a PDE5 inhibitor and riociguat, 24 h between sildenafil and riociguat administration or 48 h between tadalafil and riociguat administration should be observed.^37^ Gastroesophageal reflux and dyspepsia are common side effects of riociguat.^38^ However, riociguat is soluble at acidic pH levels; therefore, proton pump inhibitors and antacids such as aluminum hydroxide or magnesium hydroxide for reflux treatment decrease riociguat absorption and should be separated by at least 1 h.^39^ Riociguat is metabolized primarily by CYP1A1. Cigarette smoke induces CYP1A1 and can reduce riociguat plasma concentrations by 50% to 60%, which may warrant escalation in riociguat doses higher than 2.5 mg three times daily in patients who continue to smoke.^40^ Riociguat is also a substrate of CYP3A4 and transporter proteins P-glycoprotein and breast cancer resistance protein. Drugs with activity at multiple sites, such as ketoconazole (CYP3A4 and P-glycoprotein inhibitor), will increase riociguat plasma concentration and may require a lower dose at initiation of therapy.

## The Prostacyclin Pathway: Epoprostenol, Iloprost, Treprostinil, and Selexipag

Prostacyclin, or prostaglandin I_2_, is a metabolite of endogenous arachidonic acid with potent vasodilator, endothelial cell proliferation inhibition, and anti-platelet aggregation properties.^10^ In patients with PAH, the decrease of prostacyclin synthase results in less circulating prostacyclin, or prostaglandin I_2_, and causes disruption of vascular homeostasis.^41^ The available Food and Drug Administration-approved therapies that target the prostacyclin pathway include three prostacyclin, or prostaglandin I_2_, analogs (epoprostenol, iloprost, and treprostinil) and one prostacyclin receptor agonist (selexipag). Epoprostenol and iloprost are not subject to CYP450-mediated drug-drug interactions because of the route of administration and metabolism. Epoprostenol is hydrolyzed rapidly at neutral pH in blood and is subject to enzymatic degradation. Iloprost is metabolized primarily via β-oxidation to a pharmacologically inactive metabolite. Concomitant use of antihypertensive agents, diuretics, or vasodilators with prostacyclin agents may lead to increased risk of symptomatic hypotension (Table 4). Nonsteroidal antiinflammatory drugs, selective serotonin reuptake inhibitors, selective norepinephrine reuptake inhibitors, antiplatelet therapies (such as aspirin or clopidogrel), and anticoagulants may increase risk of bleeding because of additive antiplatelet properties.

Of the available prostacyclin pathway agents, treprostinil diethanolamine and selexipag are subject to potential drug-drug interactions mediated by hepatic CYP450 enzymes, primarily CYP2C8.^42^ It has not been determined if IV, subcutaneous, and inhaled treprostinil also are subject to CYP450 enzyme interactions; however, the data from the oral formulation have been extrapolated to the alternative formulations. Treprostinil diethanolamine is metabolized primarily by CYP2C8 and, to a lesser extent, by CYP2C9. Gemfibrozil, a potent CYP2C8 inhibitor, increases treprostinil concentrations twofold when coadministered. It is recommended to reduce the starting dose of treprostinil diethanolamine to 0.125 mg twice daily and to increase it by 0.125-mg twice-daily increments not more frequently than every 3 to 4 days.^43^ Selexipag is a more sensitive substrate of CYP2C8 than treprostinil diethanolamine and shows minor metabolism via CYP3A4. Clopidogrel, a moderate CYP2C8 inhibitor, increased the selexipag metabolite by approximately threefold.^44^ The dose of selexipag should be reduced to once daily in patients receiving a moderate CYP2C8 inhibitor (eg, clopidogrel, leflunomide, and deferasirox). Gemfibrozil increased the selexipag-active metabolite 11-fold.^45^ Concomitant administration of selexipag with strong inhibitors of CYP2C8 (eg, gemfibrozil) is contraindicated.^46^ Concomitant administration with an inducer of CYP2C8 and uridine 5'-diphospho-glucuronosyltransferase enzymes (rifampin) decreases the active metabolite by 50%. When administering selexipag and rifampin, the dose of selexipag should be doubled and then reduced when rifampin is stopped.^45^ CYP2C8 is one of the lesser-known metabolizing enzymes; as more inducers or inhibitors are identified, the therapeutic effects as well as adverse effects of both treprostinil diethanolamine and selexipag should be monitored.^47^

## Vasoreactivity: Calcium Channel Blockers

Approximately 10% of patients with idiopathic or heritable PAH are vasoreactive. This is defined by a reduction in mean pulmonary artery pressure of more than 10 mm Hg to an absolute value of < 40 mm Hg with unchanged or improved cardiac output when exposed to a rapidly active pulmonary vasodilator (inhaled NO or IV epoprostenol) during right heart catheterization.^48^ These cases of vasoreactive PAH respond well to high doses of oral calcium channel blockers (CCBs) such as amlodipine, nifedipine, or diltiazem. All CCBs are metabolized by CYP3A4, which introduces opportunity for significant drug interactions if coadministered with other CYP3A4 inhibitors or inducers.^49^ Verapamil, diltiazem, amlodipine, and nifedipine also are CYP3A4 inhibitors, with verapamil and diltiazem exhibiting more potent inhibition activity.^50^ Potent CYP3A4 inhibition by diltiazem and verapamil may increase the plasma concentrations of medications that rely on this enzyme for metabolism. Verapamil has a higher drug interaction risk profile than all the CCBs as a substrate and potent inhibitor of CYP3A4 and inhibitor of P-glycoprotein. The nondihydropyridine CCBs, verapamil and diltiazem, can cause excessive bradycardia. Care should be used when these nondihydropyridine CCBs are coadministered with other medications known to cause bradycardia, including noncardiac medications such as lacosamide for seizures, sphingosine 1-phosphate receptor modulators for multiple sclerosis or ulcerative colitis, and ceritinib for non-small cell lung cancer.^46^^,^^47^

## Measures to Prevent Drug-Drug Interactions

**Figure 1:**
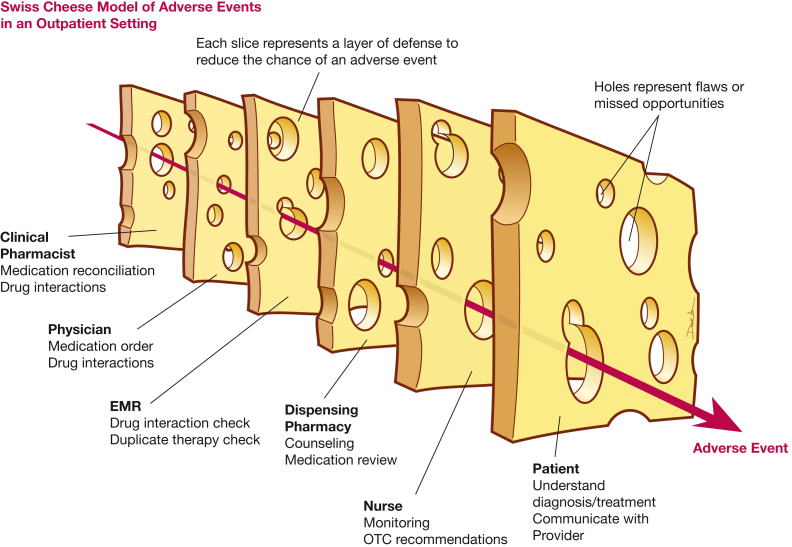
Swiss cheese model to identify opportunities for patient harm from drug-drug interactions with pulmonary arterial hypertension medications. EMR = electronic medical record; OTC = over the counter.

The prevention of potential drug-drug interactions requires multiple layers of defense. Each of these layers has potential for flaws or missed opportunities, similar to stacked slices of Swiss cheese, but these successive layers of defense provide additional protection in preventing patient harm from drug-drug interactions (Fig 1).^51^ This may start with a clinical pharmacist who initially performs a thorough medication reconciliation during the clinic visit, including dietary supplements, at every encounter and screens for drug interactions (Table 5). The PAH provider should review the medication reconciliation before making any changes to the PAH therapies. The electronic medical record can be leveraged to create alerts of potential interactions to the provider. The specialty pharmacies that dispense the PAH medications also should be screening for interactions before dispensing, because new medications could have been added by other providers. Allied health professionals such as pulmonary hypertension nurses often serve as the point of contact for patients, especially when titrating medications. Therefore, they should be knowledgeable of the interactions with commonly prescribed and over-the-counter medications used to manage side effects, such as antacids or decongestants. Allied health professionals also frequently are called by patients with COVID-19 symptoms and should be aware of drug interactions with common COVID-19 treatments and cold medications that contain decongestants. Finally, patients serve as the final layer of defense. The treatment team must educate the patient on the disease and medications being used to treat PAH, with emphasis on the drug-drug interactions that can occur. Patients should be encouraged to ask the PAH team about any new medications that outside providers may recommend or prescribe and keep an updated list of current medications. With diligence from all parties, drug-drug interactions can be minimized.

**Table 5:** Clinically Relevant Drug Metabolism Sites of PAH Medications^a^

Drug	Site of Action											
	CYP3A4		CYP2C8		CYP2C9		CYP1A1		P-glycoprotein		OATP	
	Substrate	Inducer	Substrate	Inducer	Substrate	Inducer	Substrate	Inducer	Substrate	Inducer	Substrate	Inducer
NO Pathway												
Sildenafil	X	—	—	—	X (minor)	—	—	—	—	—	—	—
Tadalafil	X	—	—	—	—	—	—	—	—	—	—	—
Riociguat	X	—	—	—	—	—	X	—	X	—	—	—
Endothelin receptor pathway												
Bosentan	X	X	—	—	X	X	—	—		—	X	—
Ambrisentan	X	—	—	—	X	—	—	—	X	—	X	—
Macitentan	X	—	X	—	X	—	—	—		—	—	—
Prostacyclin pathway												
Treprostinil diethanolamine	—	—	X	—	—	—	—	—	—	—	—	—
Selexipag	X	—	X	—	—	—	—	—	—	—	—	—

X = known activity; — = no known activity. NO = nitric oxide; PAH = pulmonary arterial hypertension. (Adapted from Ghofrani et al. Drug interactions in pulmonary arterial hypertension and their implications. US Cardiology. 2009;6(2):101-106.)

a This chart does not include all CYP activities involved with PAH medications. Above are CYP interactions identified in the current literature to have clinically significant effects. Weak interactions were omitted. See updated official prescribing information for each compound.
